# Perceived Stress and Life Stressors in Adults With and Without Fibromyalgia

**DOI:** 10.1093/geroni/igab046.2350

**Published:** 2021-12-17

**Authors:** Ha Nguyen, Courtney Buck, Barbara Cherry, Laura Zettel-Watson

**Affiliations:** California State University, Fullerton, Fullerton, California, United States; California State University, Fullerton, Fullerton, California, United States; California State University, Fullerton, Fullerton, California, United States; California State University, Fullerton, Fullerton, California, United States

## Abstract

Fibromyalgia (FM) is a widespread chronic pain condition often accompanied by comorbid conditions, such as depression, which may impact perception of stress severity. The current study examined perceived stress and life stressors in adults ages 50 and older with and without FM. It was hypothesized that individuals with FM and/or depression would subjectively rate stressors as more severe than those without. Ninety-four participants (52% with FM, 78% female) aged 50 to 93 (M = 67.72, SD = 9.26) were administered the Perceived Stress Scale (PSS) to measure perception of stress and an updated version of the Social Readjustment Rating Scale (SRRS) to assess stressors (i.e., major life events). The difference between the SRRS pre-determined values and participants' subjective ratings was calculated. Difference scores indicated that self-reported severity exceeded standardized values. Hierarchical regression analyses revealed that older adults and men were less likely to report exaggerated stress severity. Controlling for age and gender, individuals with FM were significantly more likely to report stress severity far above standardized severity scores. Both depression and chronic pain impact stress ratings, but when controlling for the former, FM impact was no longer significant, suggesting that the impact is significantly greater for depression. Results also found a significant interaction between FM status and depression for perceived stress, but not for life event stressors, which may further emphasize the distinctions between the two measures. The findings underline the importance of assessing different types of stress and stressors in individuals with chronic pain and other related comorbidities.

